# Preparation of alkali-activated oil shale residue-slag geopolymer-based binder using calcium carbide slag and sodium carbonate as alkali activator

**DOI:** 10.1371/journal.pone.0317341

**Published:** 2025-01-31

**Authors:** Chenchen Luo, Bo Zheng, Yi Li, Qiao Yang

**Affiliations:** 1 School of Civil Engineering, Wuhan Huaxia University of Technology, Wuhan, China; 2 School of Civil Architecture and Environment, Hubei University of Technology, Wuhan, China; 3 School of materials science and engineering, Huazhong University of Science and Technology, Wuhan, China; Instituto Federal do Espírito Santo: Instituto Federal de Educacao Ciencia e Tecnologia do Espirito Santo, BRAZIL

## Abstract

Based on the reuse of industrial solid wastes such as calcium carbide slag, slag, and oil shale residue, this work utilizes oil shale residue and slag as precursor materials, calcium carbide slag and sodium carbonate as alkaline activator to prepare a geopolymer-based binder. Mix proportion experiments of the geopolymer-based binder with varying oil shale residue contents were designed. Chemical composition and microstructure of the alkaline-activated oil shale residue-slag geopolymer-based binder system were analyzed using X-ray Fluorescence Spectrometer (XRF), X-ray Powder Diffractometer (XRD), and Scanning Electron Microscope (SEM). Meanwhile, the fluidity and mechanical properties of the material were also investigated. The results indicate that as the oil shale residue content increases, the fluidity and 3-day compressive strength and splitting tensile strength of the filling slurry gradually decrease, while the 28-day strength first rises and then falls, reaching a maximum when the oil shale residue content is 20%. When the oil shale residue content is below 20%, the hydration product content gradually increases, and the microstructure tends to be denser. However, as the oil shale residue content further increases, the hydration product gradually decreases, and the microstructure becomes poorer. Therefore, the optimal oil shale residue content is 20%. The geopolymer-based binder system prepared in this paper is a low-carbon, environmentally friendly, and high-performance material system, its research and development are of great significance to the human society.

## 1. Introduction

Cement is the most widely used binder in construction engineering with the largest consumption [1,2]. Ordinary Portland cement is widely used in the field of construction engineering because of its stable performance, low pollution and high yield [3]. However, the “two grinding and one burning” process in the production of Portland cement consumes a vast amount of natural resources while emitting a significant quantity of pollutants such as CO_2_ and SO_2_, accounting for more than 7% of the total global carbon emissions [4]. In recent years, there has been a growing body of research focusing on alkali-activated geopolymer-based binder. The raw materials of alkali excitation gelling materials are mostly industrial wastes (such as slag, steel slag, fly ash), and the preparation process is simple, only need to grind the raw materials, without “two grinding and one burning” like cement production. Therefore, less energy consumption, low production cost, less harm to the environment, alkali excitation gel material as an environmentally friendly material, its research and development is of great significance to human society [5–7].

Alkali-activated geopolymer-based binder, primarily composed of alkali activator and geopolymer-based binder, exhibit superior mechanical and durability properties. Their production process emits less than 40% of the CO_2_ emissions generated by traditional portland cement [8], making them one of the environmentally friendly and green geopolymer-based binder system [9,10]. Currently, the types of alkali activator mainly include hydroxides (Ca(OH)_2_), carbonates (Na_2_CO_3_), silicates (Na_2_SiO_3_·9H_2_O), phosphates (Na_3_PO_4_), and others [11]. The primary geopolymer-based binder include slag, oil shale residue, fly ash, etc. Gong et al. [12] prepared a novel geopolymer-based binder using sodium phosphate as the alkali activator and red mud-slag as the geopolymer-based binder, and founded that Sodium phosphate retards the setting and hydration of materials, and greatly decreases the heat evolution of materials during hydration. It is an effective retarder. Zhan et al. [13] explored the compressive strength and shrinkage properties of composite geopolymer-based binder with varying slag contents, using Metakaolin-Slag as the geopolymer-based binder and water-glass solution as the alkali activator. The composite geopolymer-based binder demonstrated excellent mechanical properties. Clearly, alkali-activated geopolymer-based binder are effective alternatives to ordinary Portland cement [14–17]. However, the high cost and environmental pollution associated with alkali activator in alkali-activated geopolymer-based binder have become research hotspots, focusing on how to reasonably reduce the dosage of alkali activator and select suitable activators.

Calcium carbide slag is an industrial solid waste with low utilization, mainly composed of calcium oxide. Sodium carbonate, a strong base and weak acid salt, has a lower pH value compared to sodium hydroxide. The use of calcium carbide slag combined with sodium carbonate as a composite alkali activator can not only increase the utilization rate of industrial solid waste but also reduce costs and is more environmentally friendly. Li et al. [18] studied the feasibility of calcium carbide slag replacing ordinary Portland cement as alkali excitation agent to stimulate the slag to stabilize the silt soil. The results showed that under the optimal coordination ratio, the strength of calcium carbide slag was 2 ~ 4 times higher than that of ordinary Portland cement; Gao et al. [19] used calcium carbide slag as the auxiliary activator to accelerate the Na_2_CO_3_ activated slag cement. The results showed that the addition of calcium carbide slag promoted the hardening of the slurry. Compared with ordinary Portland cement, the production of such calcium carbide slag modified Na_2_CO_3_ activated slag cement saved 94%, 87% and 20% in terms of CO_2_ emission, energy consumption and cost, respectively. This suggests that calcium carbide slag and sodium carbonate composite alkali activator are highly promising raw materials for alkali-activated geopolymer-based binder. There have been many previous studies in composite geopolymer-based binder. Zhang et al. [20] prepared the new alkali-activated materials from oil shale slag, refined high grain slag and fly ash. They found that alkali-activated materials has dense microstructure, high early strength, good mechanical and working performance. With the increase of maintenance life, there is no trend of degradation strength. Du et al. [21] with fly ash as a modifying agent, to modify high slag preparation slag cotton, realize the comprehensive utilization of industrial scattered solid waste, at the same time measured the temperature-dependent viscosity, examined the amount of fly ash on the viscosity, fluidity, particle migration activation energy, slag structure, and obtained the optimal amount of fly ash is 15%. It is evident that oil shale residue and slag are promising geopolymer-based binder, yet research on the use of oil shale residue-slag as a composite geopolymer-based binder is relatively scarce.

The above studies did not consider the influence of the mix ratio of composite geopolymer-based binder on the properties of alkali-activated geopolymer-based binder. Given the abundant production of slag and oil shale residue, utilizing these two materials to prepare alkali-activated geopolymer-based binder can not only effectively address the environmental issues arising from the accumulation of solid waste but also open up new research avenues for oil shale residue. In this study, carbide slag and sodium carbonate were selected as composite alkali activators, and oil shale residue-slag were utilized as alkali-activated materials to prepare alkali-activated composite geopolymer-based binder. For various mixing ratios of the alkali-activated oil shale residue-slag composite geopolymer-based binder, X-ray Fluorescence Spectrometry (XRF) and X-ray Powder Diffractometry (XRD) were employed to analyze the chemical compositions and particle size distributions of slag, carbide slag, and oil shale residue. Mini-slump spread tests and mechanical performance test were conducted, and Derivative Thermogravimetry (DTG) was utilized to measure the mass changes of hydration products. Mercury intrusion method was performed to analyze the micro-pore structure, and Scanning Electron Microscopy (SEM) was used to observe the micro-morphology of hydration products. Based on the experimental results, the optimal mixing ratio was determined through analysis of slurry fluidity, compressive strength, and microstructure. The findings of this study offer theoretical support for the regulation of alkali-activated geopolymer-based binder using composite geopolymer-based binder, which can facilitate the development and application of high-performance, low-cost, and environmentally friendly novel green cementitious materials, promoting the green utilization of resources.

## 2. Raw materials and experimental methods

### 2.1. Raw materials

**Fig 1:**
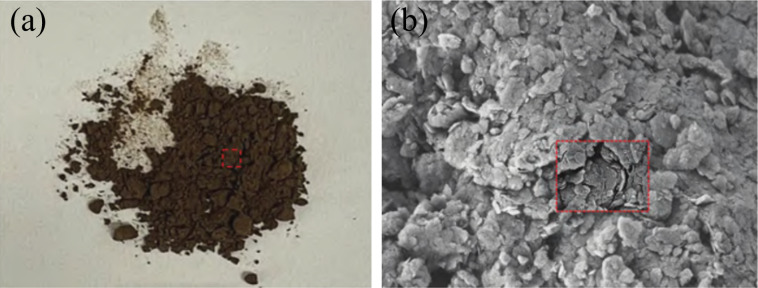
The morphology of the oil shale slag. (a)macroscopic (b) microcosmic.

The slag and carbide slag were obtained from a material company in Liaoning, China. The oil shale residue was sourced from the Fushun Open-pit West Mine in Liaoning, China. The tailings were dried to a constant weight at 105°C, and the oil shale residue underwent crushing, grinding, and calcination treatments [22], as shown in Fig 1. The oil shale residue is dark brown and the surface is covered by a large number of scales.

The chemical compositions of the three raw materials were analyzed using a X-ray fluorescence spectrometer. The XRF is produced by Panaco, The Netherlands. The model is Axios mAX with maximum power 4 Kw, maximum current 160 mA. The XRF is used to characterize the oxide content of the raw materials used in the study, and the resulting chemical composition table is shown in Table 1. The main components of the slag are SiO_2_, Al_2_O_3_, and CaO, while carbide slag is primarily composed of CaO. The oil shale residue is mainly composed of SiO_2_ and Al_2_O_3_, the total amount is more than 80%. According to the national standard GB/T 203-2008 “Granulated Blast Furnace Slag for Use in Cement,” the slag is classified as high-quality acidic slag.

**Table 1:** Chemical composition of raw materials/%.

Raw materials	SiO_2_	Al_2_O_3_	CaO	MgO	Fe_2_O_3_	Na_2_O	K_2_O
**Calcium carbide slag**	3.05	0.81	94.84	0.06	0.77	0.05	–
**Slag**	33.66	15.60	39.62	6.78	0.40	0.06	0.42
**Oil shale slag**	62.07	22.53	0.78	0.94	8.12	0.05	1.21

**Fig 2:**
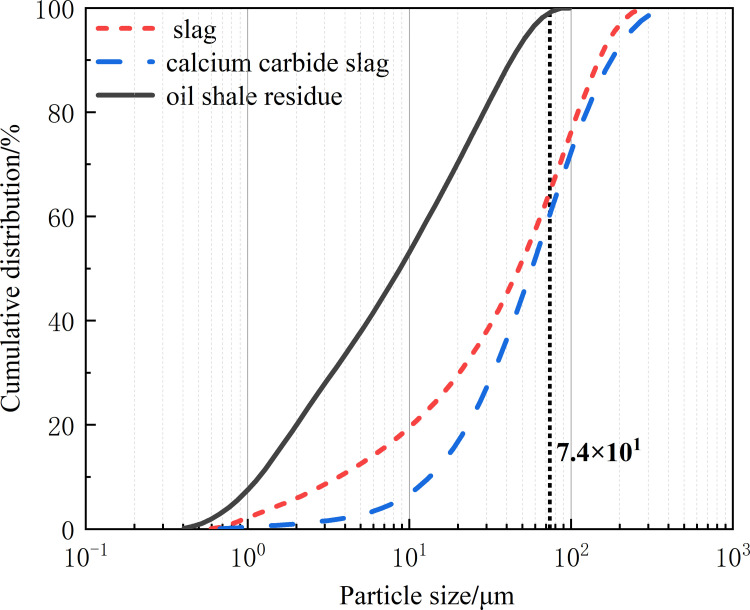
Particle size distribution of the raw materials.

**Fig 3:**
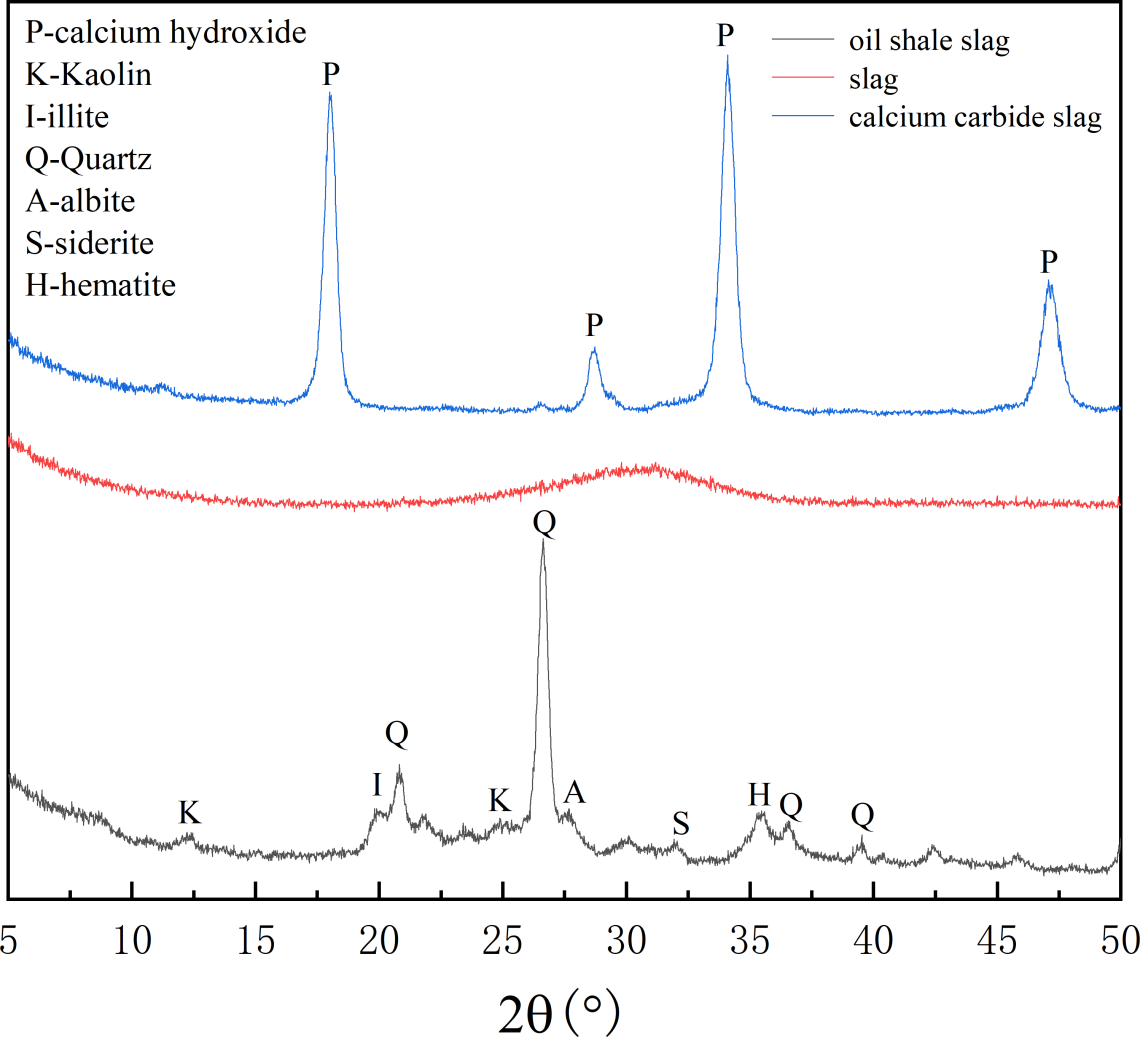
Mineral composition analysis of raw materials.

The particle size distribution of the raw materials was tested and characterized using a JL-1155 laser particle size analyzer, and the test results are shown in Fig 2. Its particle size distribution in 0.52 µm ~ 74 µm, the average particle size of 50 µm, belonging to the powder particle, the specific surface area of large [23], with a relatively high reactivity during processing. The maximum particle size of calcium carbide slag and slag is greater than 74 µm. The mineral composition was tested and analyzed using a UItima IV X-ray diffraction analyzer, as shown in Fig 3. The main component of oil shale slag is quartz, which contains a small amount of clay minerals such as kaolin and illite, which have a certain number of glass phase in the structure, so the hydration reaction can occur in alkaline solution [24]. The main component of calcium carbide slag is calcium oxide. The aggregate used in the mortar is ISO standard sand, and the mixed water used in the experiment is tap water.

### 2.2. Mix design methods and specimen preparation

Based on the national standard GB/T 17671-2021 “Test Method for Strength of Cement Mortar (ISO Method),” the standard sand weighs 1350 g, and the water weighs 225 g. The total mass of slag and oil shale residue was determined to be 450 g. According to previous literature research [25], when the concentration of sodium hydroxide (the ratio of solid sodium hydroxide to slag weight) is 5%, the compressive strength of alkali-activated slag is highest. Therefore, in this study, a mixture of 22.5 g of calcium carbide slag and sodium carbonate is used to replace sodium hydroxide. Based on the chemical reaction equation (1), the molar ratio of calcium carbide slag to sodium carbonate is 1:1, resulting in the respective masses of calcium carbide slag and sodium carbonate being 9.25 g and 13.25 g. While maintaining the total mass of slag constant, the proportions of slag and oil shale residue in the raw materials were varied, as detailed in Table 2. The doping amounts of oil shale residue in N0 to N4 were 0%, 10%, 20%, 30%, and 40%, respectively.

**Table 2:** Design of alkali activated mortars/ kg·m^−3^.

Number	Slag/g	Oil shale residue/g	Standard sand/g	Calcium carbide slag/g	Na_2_CO_3_/g	Water/g
**N0**	450	0	1350	9.25	13.25	225
**N1**	405	45	1350	9.25	13.25	225
**N2**	360	90	1350	9.25	13.25	225
**N3**	315	135	1350	9.25	13.25	225
**N4**	270	180	1350	9.25	13.25	225


$$\text{CaO}+{\text{H}}_{\text{2}}\text{O}+{\text{ Na}}_{\text{2}}{\text{CO}}_{\text{3}}\to \text{NaOH}+{\text{CaCO}}_{\text{3}}$$ (1)

**Fig 4:**
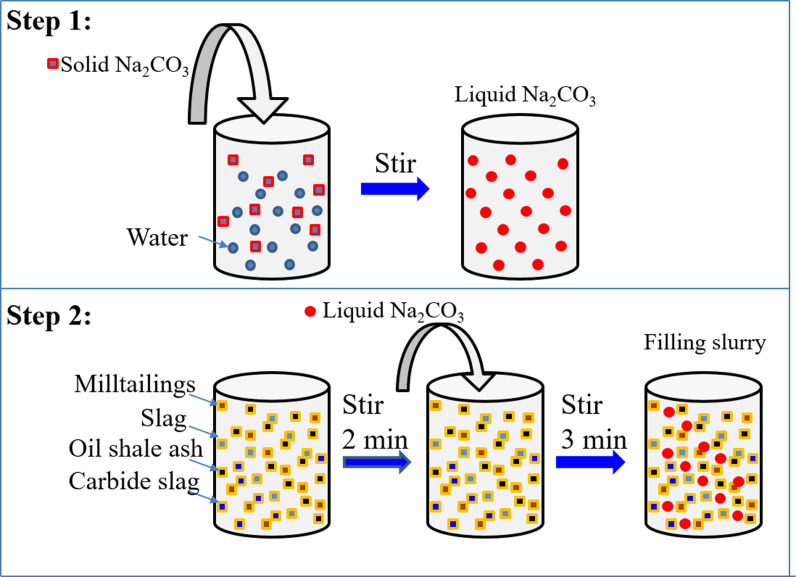
Schematic diagram of experimental scheme.

Fig 4 illustrates the schematic diagram of sample preparation. Sodium carbonate was added to water and stirred thoroughly until no solid particles remained in the beaker. The tailings, slag, oil shale residue, and calcium carbide slag dry materials were mixed for 2 minutes using a JJ-5 mortar mixer. Immediately afterward, the prepared sodium carbonate solution was poured into the mixing container and stirred for an additional 3 minutes to obtain a fresh filling slurry.

### 2.3. Mini-slump extension test

**Fig 5:**
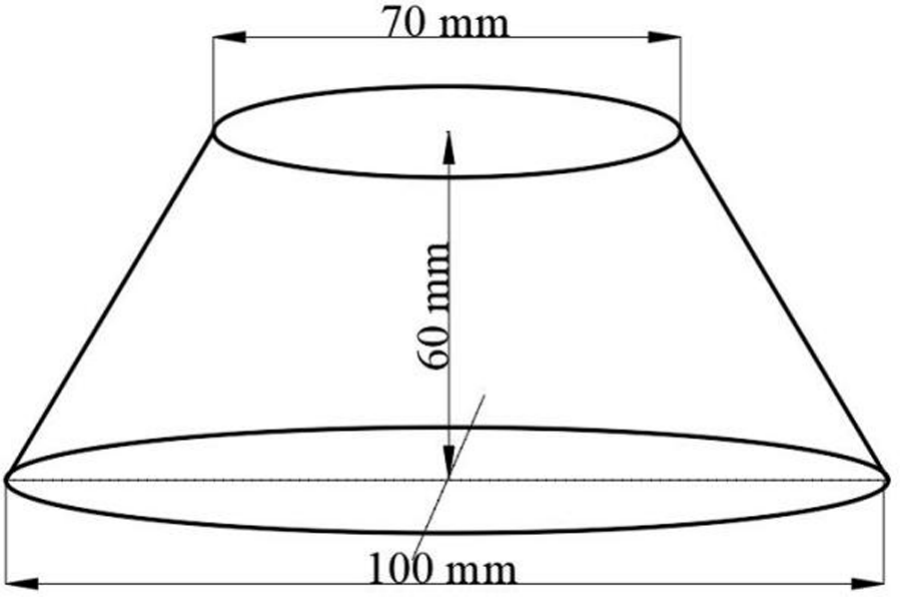
Mini-slump extension test cylinder.

This study employed the mini-slump flow test method proposed by Okamura and Ouchi [26]. After mixing the slurry, a portion of it was removed from the mixing container and poured into a steel mold with an upper inner diameter of 70 mm, a lower inner diameter of 100 mm, and a height of 60 mm. The surface of the slurry was leveled with a steel ruler, and the steel mold was then removed vertically, allowing the slurry to flow freely. The height of the descending slurry was measured with a ruler to the nearest 0.1 mm. Two steel rulers were then placed perpendicular to each other along the slurry. When the collapsed slurry formed a circular disk, the diameters in two directions were measured to the nearest 1 mm, and the average of these two diameters was taken as the spread. For irregular circles, measurements were taken twice along the diagonal directions, and their average was used. The dimensions of the mini-slump flow test apparatus are shown in Fig 5.

### 2.4. Mechanical performance test

The slurry was poured into a cylindrical mold with dimensions of Φ50 mm×100 mm, sealed, and cured in a curing chamber at a temperature of 20±2°C and a relative humidity of 95±2% until the target age (3 days, 28 days). After reaching the target age, using CMT5105 electronic universal tester, use strain control method for loading, carry out unlimited compressive strength test and split tensile strength test, and control the loading rate of 1 mm/min. Three parallel trials were performed for the same age period and the same ratio, and the results were averaged.

### 2.5. Detection of the hydrated products and the microscopic pore structures

After curing the filling body specimens for 28 days, break the test piece with a crushing hammer, and then use scissors to take a size of about 1 cm^3^ at the center of the test block. These fragments were immersed in isopropanol for 24 hours to stop hydration reactions [27,28], then dried at 40±2°C for 8 hours. Thermogravimetric analysis (TGA) was performed on 20–30 mg samples using a STA409PC integrated thermal analyzer under a nitrogen atmosphere, heating from room temperature (30°C) to 1000°C at a rate of 15°C/min. After cooling the remaining filling body specimens to room temperature, micro-pore structure testing was conducted using an AutoPore IV-9500 mercury intrusion porosimeter. The microscopic morphology of the fillings was observed by MIRA3 LMH scanning electron microscope.

## 3. Results and discussion

### 3.1. Flowability

**Fig 6:**
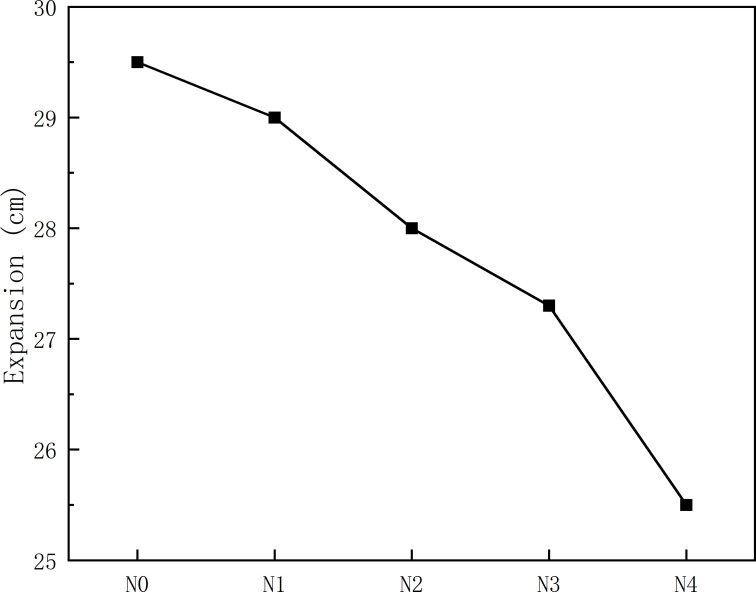
Flow spread of slurry N0 ~ N4.

Fig 6 presents the results of the variation in the spread of the slurry with different amounts of oil shale residue. The extension range of oil shale slag-slag gel material system is 255 ~ 295 mm, which is much higher than Li et al. [29]. The extension range of NaOH alkali excitation gel material of fly ash and ore powder with different mass ratio is 50 ~ 130 mm, so the rheological energy of oil shale slag-slag gel material system is far better than that of fly ash-ore powder gel material system.

As shown in Fig 6, the spread of the slurry gradually decreases with an increase in the oil shale residue content, indicating that oil shale residue reduces the fluidity of the slurry. According to the particle size analysis of the raw materials in Fig 2, oil shale residue contains a large amount of fine particles, with the content of particles smaller than 74 μm almost reaching 100%. Fine particles have a large specific surface area and good water retention properties, which lead to a reduction in the amount of water available for lubrication in the slurry. Furthermore, compared to slag, which is primarily composed of vitreous materials, the high water absorption rate of the clay in oil shale residue also results in a decrease in the free water content in the slurry. The reduction in free water content increases the probability of particle collisions within the slurry and leads to increased friction between particles, consistent with the report by Wang et al. [30]. They studied the rheological characteristics of Coal gangue-fly ash slurry, and found that the slurry fluidity was closely related to the concentration of fly ash slurry. The rate of decrease in the spread of the slurry with increasing oil shale residue content was greater than that for the 0–10% range, indicating that the adverse effect on slurry fluidity became more pronounced with higher oil shale residue content.

### 3.2. Mechanical performance analysis

**Fig 7:**
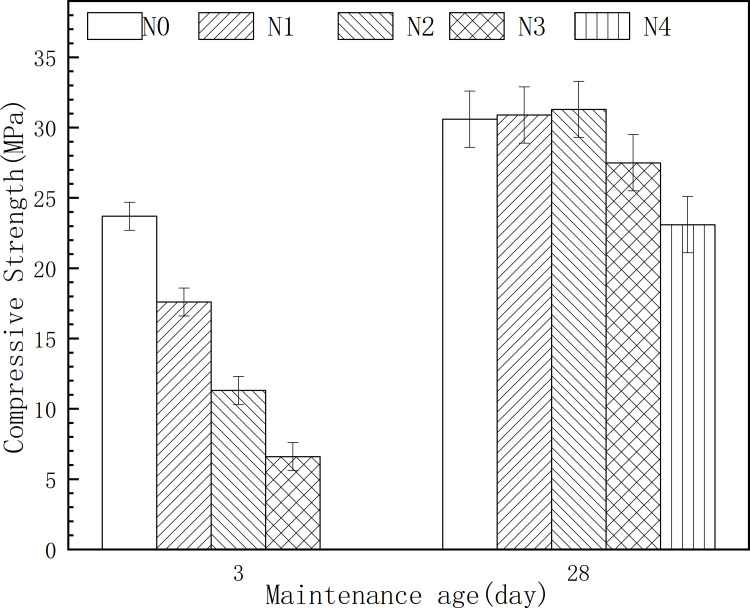
Effect of OSR content on the compressive strength.

Fig 7 shows the results of the changes in the compressive strength of the backfill at 3 days and 28 days with different oil shale residue contents. As shown in Fig 7, The initial compressive strength of the filling body was 23.7 MPa in 3 days and 30.6 MPa at 28 days. With the increase of oil shale slag, the strength of the filling body gradually decreased, decreasing to 6.6 MPa when the mixing amount was 30%, or 72.1%, until the mixture was 40% to 0. However, the intensity at 28 days first increased and then decreased, and reached the highest point at 31.3 MPa at 20% incorporation. Yang et al. [31] studied the mechanical properties of polymer grouting material of steel fiber at composite nanoAl_2_O_3_ at different amounts. The 3 days compressive strength range is between 7.5 ~ 12.5 MPa, and the 28 days compressive strength range is between 12.5 MPa and 21 MPa. Therefore, the compressive strength of filling body is much greater than that of polymer grouting material at composite nano Al_2_O_3_.

The main reason for the decrease of strength of filling at the maintenance age of 3 days is that the activity of oil shale slag is much lower than that of slag. As shown in Fig 3, slag is primarily composed of amorphous vitreous materials with high reactivity, enabling early hydration reactions to produce gelled phases such as calcium silicate hydrate, thereby providing early strength. In contrast, oil shale residue is dominated by inert minerals such as quartz, and although it contains clay minerals like illite and kaolinite, which exhibit some reactivity after calcination, its overall reactivity is still much lower than that of slag, resulting in a loss of early strength. When the oil shale residue content reached 40%, no 3-day compressive strength was observed due to the failure of the specimen to set, indicating that the oil shale residue content should be kept below 30%.

**Fig 8:**
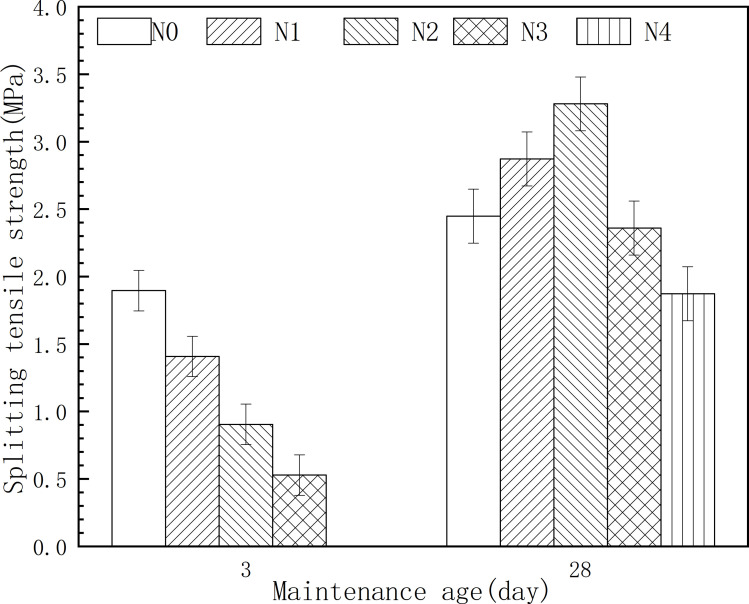
Effect of OSR content on the Splitting tensile strength.

Fig 8 shows the results of the changes in the splitting tensile strength of the backfill at 3 days and 28 days with different oil shale residue contents. As shown in Fig 8, with the increase of oil shale residue, the tensile strength of filling 3 days gradually decreased, and the tensile strength of 28 days increased first and then decreased, reaching the highest point at 20%. Among them, when the amount of 28 days oil shale slag increased from 0% to 10%, the splitting tensile strength increased from 2.448 MPa to 2.872 MPa, an increase of 17.3%. When the amount of oil shale slag reaches 30%, the tensile strength of packing is reduced to 0.58 MPa. The splitting tensile strength of the filling body increases with the growth of age, and the curing 3 days splitting tensile strength is 1.408 MPa, and when the curing continues to 28 days, the splitting tensile strength increases by 104%, that is, 2.872 MPa. This strength is much higher than the 28 days splitting tensile strength value of 1.2 MPa of curing construction muck using cement and calcium bentonite curing agent by Guo et al. [32].

It can be seen that the split tensile strength coincides with the law of the unrestricted compressive strength, and only has a certain difference in value. Unlike the 3 days strength, the 28 days strength of the hardened backfill showed a trend of increasing first and then decreasing, suggesting that the incorporation of oil shale residue is beneficial to the development of compressive strength when the content is less than 20%. This is mainly due to the pozzolanic activity of oil shale residue being effective at 28 days. Kaolinite in oil shale residue can be converted into metakaolin after calcination, which has high pozzolanic activity. It can react with residual calcium hydroxide in carbide slag or calcium hydroxide generated by the hydration of slag to form C-(A)-S-H, reducing the interface transition zone and producing more gelled phases, thereby promoting the strength development of the hardened backfill. Combining the results of the slurry spread in Fig 6, it can be concluded that the optimal oil shale residue content is 20%. In this study, the 3 days and 28 days compressive strengths of the alkali-activated composite cementitious material were 12.5 MPa and 33.3 MPa, respectively, meeting the standards of GB 175-2023 “General Portland Cement” for slag cement with a strength grade of 32.5, with a 3 days compressive strength ≥ 12 MPa and a 28-day compressive strength ≥ 32.5 MPa, satisfying usage requirements.

### 3.3. Thermogravimetric analysis

**Fig 9:**
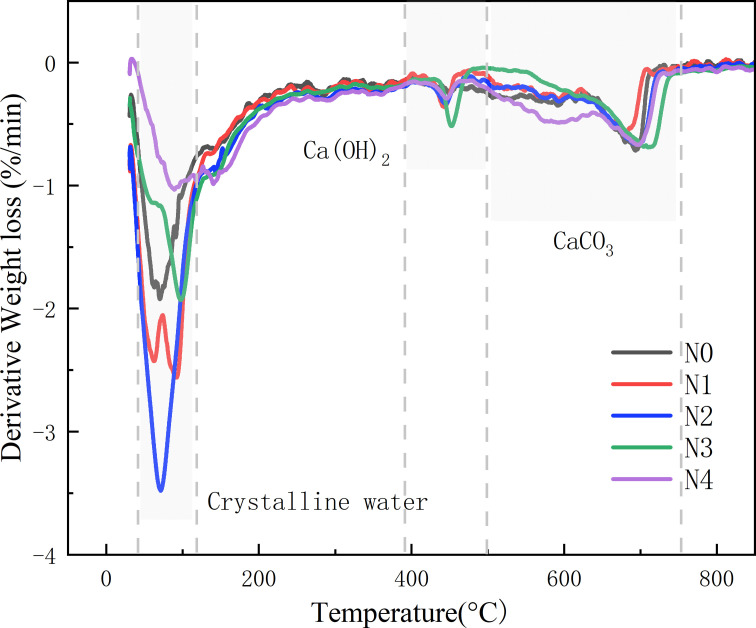
Effect of OSR content on the DTG curves of hydration products.

The Derivative Thermogravimetry (DTG) curve of the filling specimens after 28 days of curing is shown in Fig 9. It can be observed from Fig 9 that there is a most pronounced downward peak between 50°C and 100°C, indicating significant weight loss within this temperature range. According to the existing literature of [33], the loss of samples between 50°C and 120°C is mainly due to the loss of crystalline water in the hydration products, and the loss rate of N0–N4 samples are 1.9%/min, 2.6%/min, 3.4%/min, 1.8%/min, 1.0%/min, respectively. It can be seen that the weight loss rate of N2 is the largest, 3.4%/min, which is much higher than that of Chang et al. [34] Using calcined layered double hydroxide (CLDH) and Na_2_CO_3_ to stimulate the blast furnace slag-fly ash based cement material, the weight loss rate of the sample between 50°C and 150°C is 1.6%/min, the greater the weight loss in this temperature segment, the greater the amount of hydration products in the filling body. Between 400°C and 500°C, the primary decomposition is that of Ca(OH)_2_ [35], while between 600°C and 750°C, it is mainly the decomposition of CaCO_3_ [36]. When the amount of oil shale slag is 20%, the sample weight loss is most obvious, which indicates that the hydration product content of the hardened filling is the highest under this amount, which also verifies the change of the compressive strength of the hardened filling at 28 days.

### 3.4. Microscopic pore structure

**Fig 10:**
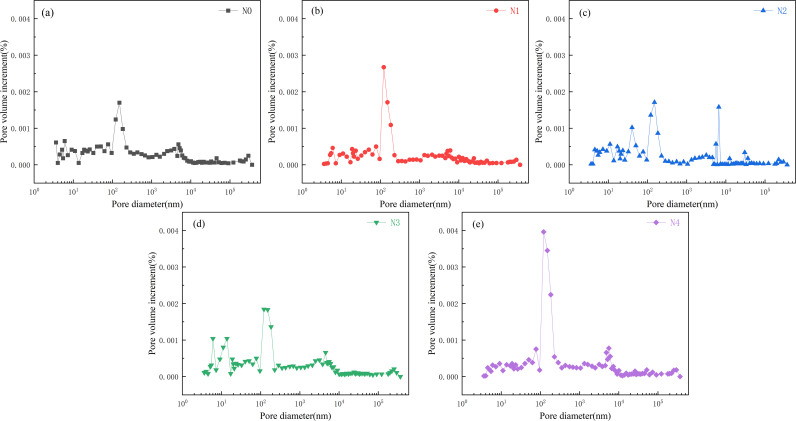
Effect of oil shale residue content on the incremental pore volume of hardened filler.

**Fig 11:**
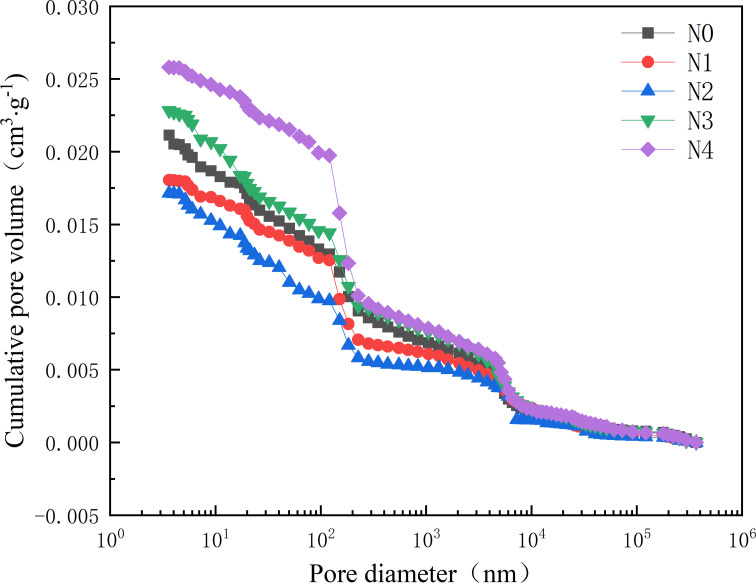
Effect of oil shale residue content on the cumulative pore volume of hardened filler.

Figs 10 and 11 respectively present the incremental and cumulative distributions of micro-pore volume in the hardened filling after 28 days of curing. As the oil shale ash content increases, the cumulative pore volume of the hardened filling initially decreases and then increases, suggesting that the microstructure first becomes denser and then degrades. The optimal microstructure with the lowest micro-pore content is achieved at an oil shale ash content of 20%. The reason for the variation in micro-pore volume distribution is similar to the trend in compressive strength. The incorporation of oil shale ash consumes calcium hydroxide in the mixture and forms more hydrated calcium silicate or hydrated calcium aluminosilicate gels, which effectively fill the micro-pores [37]. Additionally, as shown in Fig 2, the particle size distribution of oil shale ash is much smaller than that of slag, indicating a favorable micro-filling effect. However, when the oil shale ash content exceeds 20%, the reduced amount of slag available for hydration leads to a decrease in hydration products, and the pozzolanic reaction of oil shale ash cannot compensate for this loss, ultimately resulting in microstructural degradation. Ma et al. [38] use fly ash-slag as cementing material, sodium silicate water glass and sodium hydroxide as alkaline activator, the cumulative pore volume of 28 days is 0.017 ~ 0.03 cm^3^/g. In this paper, the cumulative pore volume of 28 days obtained by the preparation of calcium carbide slag-sodium carbonate as alkali excitation agent is 0.017 ~ 0.026 cm^3^/g, which is consistent with the reported value and provides a way for the preparation of new cemelling materials.

### 3.5. Micro-morphology of hydration products

**Fig 12:**
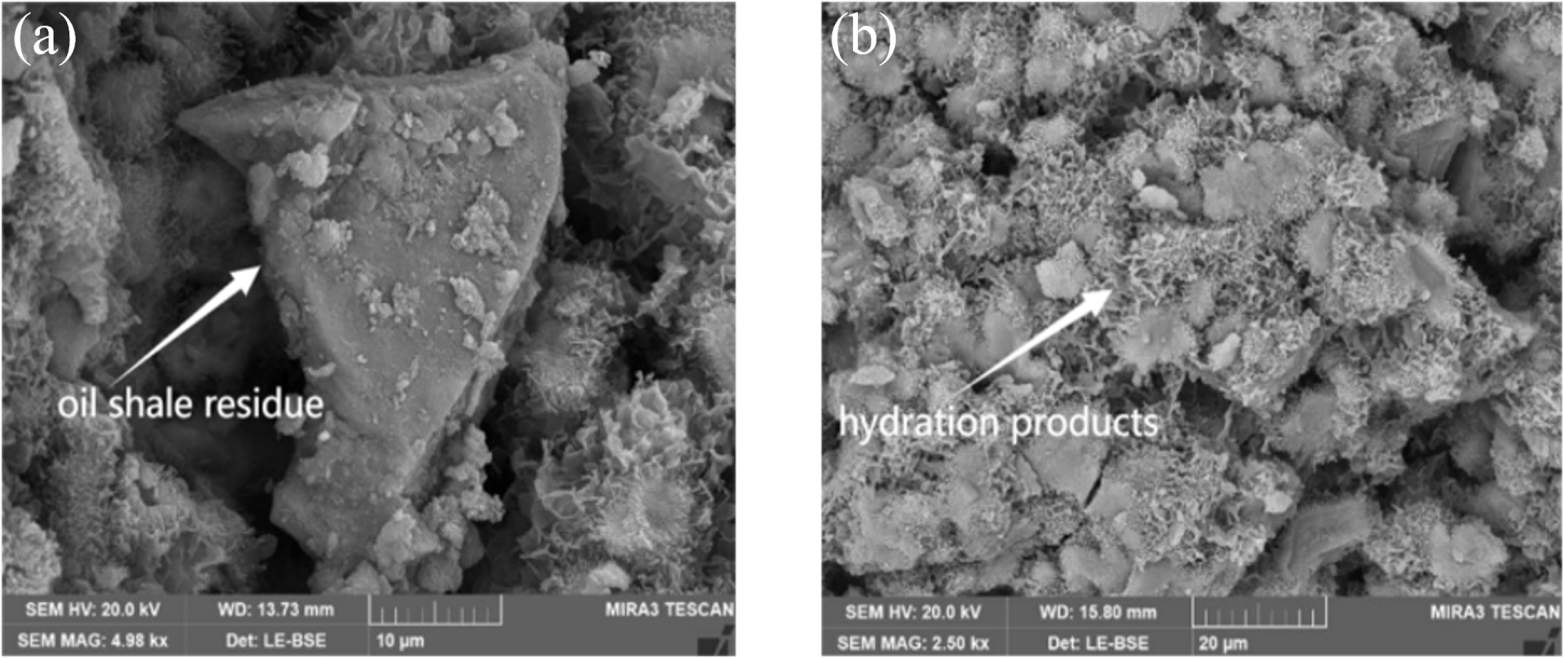
Micro-schematic diagram of hydration products of N2. (a) unhydrated oil shale residue particle (b) morphology of hydration products.

Fig 12 illustrates the microscopic morphology of hydration products in specimen N2 after 28 days of curing. From Fig 12(a), intact oil shale residue particles can be observed, indicating that the reactivity of oil shale residue is relatively low and it has not fully reacted. However, Fig 12(b) shows a large amount of reticular hydration products adhering to the unreacted oil shale residue, suggesting that oil shale residue plays a significant role in nucleation and pozzolanic activity [39]. This is because the main component of oil shale residue is SiO_2_. Yu et al. [40] pointed out that SiO_2_ has pozzolanic effect. The following reaction will occur SiO_2_ + Ca(OH)_2_ + H_2_O → Ca_5_Si_6_O_16_(OH)·4H_2_O (C-S-H gel). Enough silica-generated C-S-H can allow the hydration product growth and produce dense C-S-H gel, which is a typical effective nucleating agent [41]. While promoting the hydration of slag, it reacts with the calcium hydroxide generated from the hydration of slag to form hydrated silicates (aluminates) such as calcium silicate hydrate.

## 4. Conclusions

In this study, a backfill material consisting of oil shale residue and slag as geopolymer-based binder, sodium carbonate and carbide slag as composite alkaline activators, was prepared. The following conclusions were drawn:

(1) As the proportion of oil shale residue increases, the fluidity of the backfill material gradually decreases. This is mainly due to the higher water absorption of oil shale residue, which contains a large amount of clay and incompletely calcined organic matter. As a result, the free water content in the slurry decreases, leading to a reduction in fluidity.
(2) With an increase in the proportion of oil shale residue, the 3 days compressive strength of the hardened backfill material gradually decreases, while the 28 days compressive strength initially increases and then decreases. This indicates that compared to slag, the reactivity of oil shale residue is lower in the early stages, resulting in a loss of early strength. However, after 28 days of curing, the oil shale residue can react with the calcium hydroxide generated from hydration to form hydrated silicates (aluminates) such as calcium silicate hydrate. The optimal proportion of oil shale residue is 20%.
(3) Microscopic test results and other findings indicate that when the content of oil shale residue is less than 20%, an increase in the content leads to a denser structure in the hardened backfill material. However, with further increases in the content, the structure becomes looser. This consistency with the compressive strength results further confirms that the optimal proportion of oil shale residue is 20%.
(4) Scanning electron microscopy results reveal that the reactivity of calcined oil shale residue remains low, and it does not possess self-hydration capability. Its main roles are pozzolanic activity and nucleation effect.

The strength of the cementing material in this study meets the requirements of ordinary construction engineering, which not only contributes to the environmental protection of the construction industry, but also can be used for the material selection of temporary buildings, and opens up a new way for the resource utilization of solid waste.

## Supporting information

S1 Data Raw data. (ZIP)
